# Antimicrobial Stewardship Programmes in Community Healthcare Organisations in England: A Cross-Sectional Survey to Assess Implementation of Programmes and National Toolkits

**DOI:** 10.3390/antibiotics7040097

**Published:** 2018-11-07

**Authors:** Diane Ashiru-Oredope, Anne Doble, Mary Richard Akpan, Sejal Hansraj, Nada Atef Shebl, Raheelah Ahmad, Susan Hopkins

**Affiliations:** 1 HCAI & AMR Division, Public Health England, London NW9 5EQ, UK; anne.doble@phe.gov.uk (A.D.); s_hansraj@hotmail.com (S.H.); susan.hopkins@phe.gov.uk (S.H.); 2 Department of Clinical and Pharmaceutical Sciences, University of Hertfordshire, Hatfield AL10 9AB, UK; akpanmary02@gmail.com (M.R.A.); n.a.shebl@herts.ac.uk (N.A.S.); 3 NIHR Health Protection Research Unit in Healthcare Associated Infection and Antimicrobial Resistance at Imperial College London, Hammersmith Campus, Du Cane Road, London W12 0NN, UK; raheelah.ahmad00@imperial.ac.uk

**Keywords:** antibiotics, community hospitals, community health trusts, start smart then focus, general practice, TARGET toolkit

## Abstract

Objective: The aim of this study was to assess antimicrobial stewardship activities in Community Healthcare Organisations (CHOs) with focus on the implementation of the two national antimicrobial stewardship toolkits, TARGET (Treat Antibiotics Responsibly, Guidance, Education, Tools) and SSTF (Start Smart, then Focus). The study utilised a web-based survey comprising 34 questions concerning antimicrobial policies and awareness and implementation of antimicrobial stewardship toolkits. This was distributed to pharmacy teams in all 26 CHOs in England. Twenty CHOs (77%) responded. An antimicrobial stewardship (AMS) committee was active in 50% of CHOs; 25% employed a substantive pharmacist post and 70% had a local antibiotic policy. Fourteen of the responding CHOs were aware of both AMS toolkits, five organisations were aware of either SSTF or TARGET, and one organisation was not aware of either toolkit. Of the organisations aware of SSTF and TARGET, eight had formally reviewed both toolkits, though three had not reviewed either. Less than half of the respondents had developed local action plans for either toolkit. National guidance in England has focused attention on initiatives to improve AMS implementation in primary and secondary care; more work is required to embed AMS activities and the implementation of national AMS toolkit recommendations within CHOs.

## 1. Introduction

Antimicrobial resistance (AMR) poses a serious threat to global public health with clinical and economic implications. Antibiotic-resistant bacterial infections are associated with increased mortality compared to those without resistance [1]. The overuse and inappropriate use of antimicrobials is recognised as the major driver for the development of AMR [2].

Antimicrobial stewardship (AMS) programmes encourage the responsible use of antimicrobials through the delivery of multiple evidence-based interventions. Studies demonstrate that AMS interventions reduce excessive antibiotic prescribing in secondary care, can reduce AMR and healthcare associated infections (HCAIs), increase effective prescribing, and improve clinical outcomes for patients [3]. AMS programmes are increasingly regarded as essential to tackling AMR and safeguarding human health [4,5].

In 2013, a cross-government five-year strategy was published to tackle AMR in the UK. A key aim of this strategy was to optimise prescribing practice through the implementation of AMS programmes [6]. Two national evidence-based AMS toolkits are currently available for use by healthcare organisations in England: Start Smart, then Focus (SSTF) [7] and Treat Antibiotics Responsibly, Guidance, Education, Tools (TARGET) [8] for secondary and primary care, respectively. SSTF was produced by Public Health England (PHE) in 2011 and updated in 2015. It recommends prompt antibiotic treatment for hospital inpatients using local guidance based on local resistance patterns; advises on the documentation of route, indication, dose, duration, and 48–72 h review after initiation of antibiotic therapy; and recommends routine audits to assess adherence to AMS principles. TARGET was produced by PHE in collaboration with the Royal College of General Practitioners in 2012. The toolkit provides guidance to help general practice decide when and what antibiotics to prescribe and tools such as patient leaflets to share during consultations, as well as educational and audit materials. The implementation of these toolkits and associated audits is a criterion within the “Health and Social Care Act 2008: Code of Practice on prevention and control of infections” [9]. In addition, they are recommended as part of the National Institute for Health and Care Excellence (NICE) guidance on AMS [10]. We have previously conducted studies to assess the implementation of these toolkits in primary care (general practice) and secondary care (acute care hospitals) [11]. Both toolkits are also available for use in Community Healthcare Organisations (CHOs). However, a gap currently exists in the knowledge of antimicrobial use and AMS activities in this setting. CHOs are specialist organisations that provide community-based health services for adults and children, such as district nursing (including out-of-hours nursing), health visiting, school nursing, physiotherapy, speech and language therapy, and podiatry.

Surveillance data from 2014 showed that 74% of the antibiotics prescribed in England were in general practice (primary care), 18% were prescribed for patients in secondary care, 5% for patients in dental practices, and 3% for patients in community organisations [12]. Though prescribing within CHO settings accounts for a relatively small proportion, this setting represents an important sector within healthcare delivery.

The aim of this study was to gain an understanding of AMS activities in the community health setting with respect to the implementation of the two national AMS toolkits and to highlight where improvements may be required.

## 2. Results

**Figure 1:**
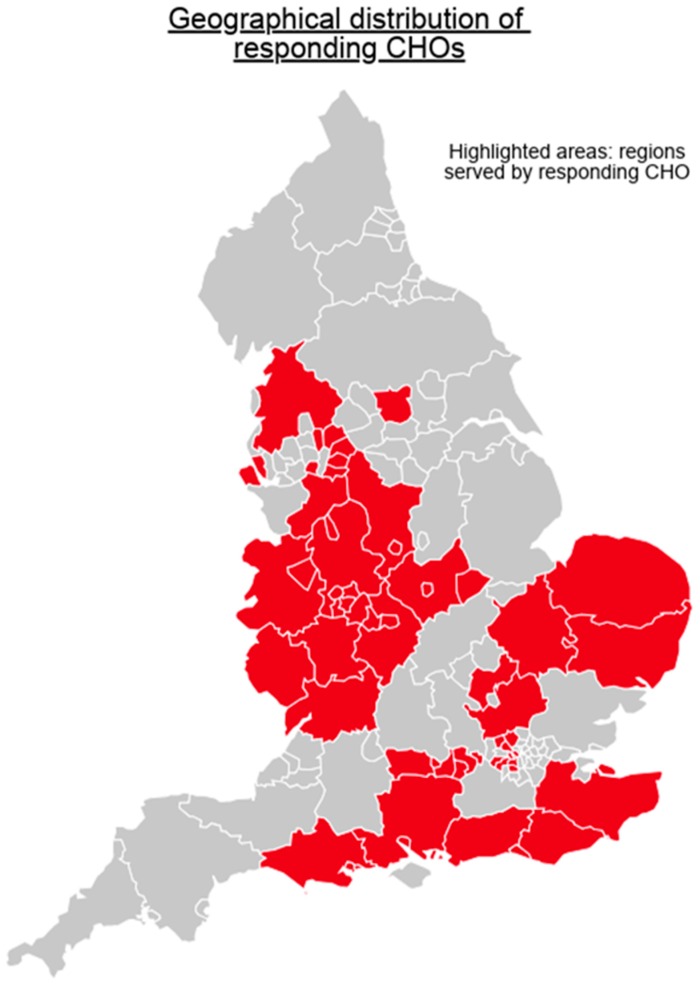
Map of England showing the locations of the Community Healthcare Organisations (CHOs) that responded to the survey by region. Figure created using Piktochart.

Twenty CHOs (77%) out of the 26 CHOs in England responded to the survey; there were responsible for managing 93 hospitals overall and serving a total population of nearly 28 million (according to population data reported within individual CHO publications). The responding CHOs were broadly located in three geographical regions: The West and North West of England (ten CHOs covering a population of nearly 15.3 million); the East of England (two CHOs covering a population of around 4 million); and London, the South East and the south coast (seven CHOs covering 7.8 million). See Figure 1 for a map showing the geographical regions served by CHOs that responded to the survey.

Ten percent (2/20) of CHOs had 10–50 beds, 20% (4/20) had 51–100 beds, 10% (2/20) had 101–200 beds, and 60% (12/20) had >200 beds. Sixty percent (12/20) of CHOs had beds for use as inpatient step-up (patients admitted from home as an alternative to acute hospital admission), 85% (17/20) had beds for inpatient step-down (patients transferred from acute hospital, for people who require additional time and rehabilitation to recover but are unable to have this provided at home), and 40% had beds (8/20) for mental healthcare. The services provided by the majority of responding community health service organisations included dentistry, palliative care, physiotherapy, podiatry, rehabilitation, sexual health, learning disabilities services, and minor injury and illness centres and services for older adults.

### 2.1. Implementation of National Toolkits

Eighteen (90%) of the responding CHOs indicated that they were aware of the primary care toolkit, TARGET, and fifteen (75%) were aware of the secondary care toolkit, SSTF. One organisation was not aware of either toolkit and fourteen were aware of both. The AMS national toolkit implementation and other AMS activities reported by the CHOs are summarised in Table 1.

### 2.2. Informal/Formal Review and Implementation of the TARGET or SSTF Toolkits between CHOs

Sixteen CHOs were both aware of the TARGET toolkit and had either formally or informally reviewed it. Of these, seven (44%) had action plans in place and ten (62.5%) had implemented audits. By comparison, the two CHOs that were aware of the TARGET toolkit but had not reviewed it (formally or informally) had neither implemented audits nor put action plans in place. In terms of the SSTF toolkit, all CHOs that were aware of the toolkit had also either formally or informally reviewed the toolkit. Of those CHOs aware of both toolkits (*n* = 14), over one third had written action plans in place (5/14) and eight (57%) had formally reviewed both toolkits. Eight CHOs had also implemented suggested audits.

### 2.3. Implementation of both TARGET and SSTF Toolkits Across CHOs with Varying Bed Numbers and Bed Types

**Figure 2:**
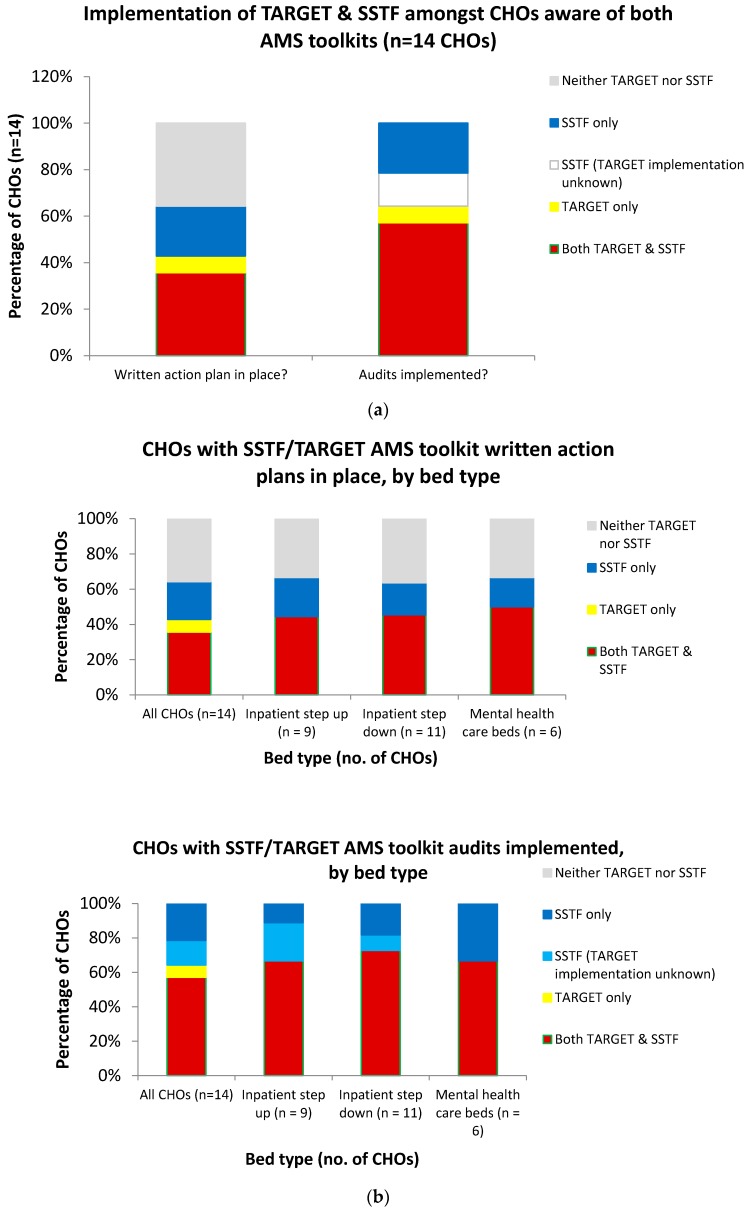

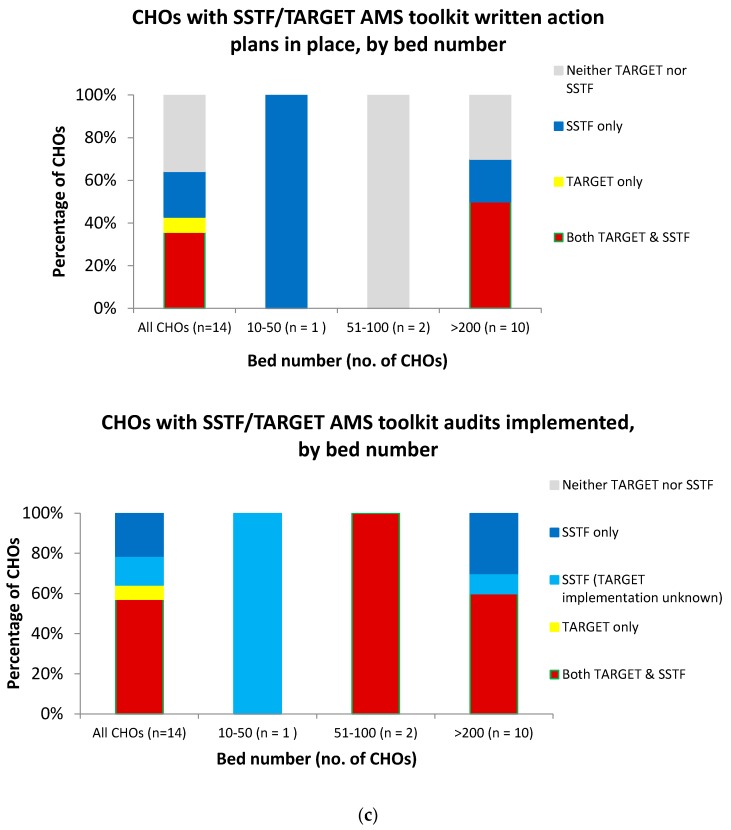
Implementation of Start Smart, then Focus (SSTF) and/or Treat Antibiotics Responsibly, Guidance, Education, Tools (TARGET) antimicrobial stewardship (AMS) toolkits amongst the CHOs that were aware of both toolkits, overall and by CHO bed type and bed number. (**a**) Presence of written action plans in place and implementation of audits across all trusts. (**b**) Presence of written action plans in place and implementation of audits by bed type. (**c**) Presence of written action plans in place and implementation of audits by bed number.

The implementation of the TARGET and SSTF toolkits amongst CHOs that were aware of the toolkits was compared according to CHO characteristics: bed number and bed type (Figure 2). Six of the ten CHOs with >200 beds had audits implemented for both SSTF and TARGET (60%); all ten had audits implemented for at least one. For the four CHOs with <200 beds, implementation of audits for both SSTF and TARGET was 50% and implementation of at least one was 75%. The five CHOs with written action plans in place for both toolkits all had >200 beds. Five further CHOs did not have written action plans in place for either TARGET or SSTF; three were for CHOs with >200 beds and two were for CHOs with <200 beds. The remaining three CHOs (one with 10–50 beds and two with >200) had action plans in place for SSTF only. With respect to bed type, there was very little difference between the numbers of those with written action plans in place or those that had audits implemented for both/neither TARGET and SSTF toolkits (Figure 2). 

### 2.4. Implementation of Both TARGET and SSTF Toolkit between CHOs with or without an AMS-Focused Pharmacy Post or Dedicated Antimicrobial Policy

Considering the fourteen CHOs that were aware of both toolkits, of the five CHOs with an AMS-focused pharmacy post, three had local action plans on an AMS for both toolkits (60%), three had implemented audits (60%), and four had dedicated antibiotics policies (80%). In a comparison of the nine without pharmacy posts, only two had local action plans (22%), five had implemented audits (56%), and seven had antibiotic policies (78%).

### 2.5. Implementation of AMS and AMR Education and Training Initiatives across CHOs

The implementation of education and training initiatives varied between the healthcare professional groups (Table 2). In 40% of the responding CHOs, prescribers (both doctors and non-medical prescribers) received AMS/AMR teaching at induction, and in 35% of CHOs this was received by all staff groups. In eight out of twenty CHOs, both doctors and pharmacists received education when starting in the organisations. There was limited access to e-learning modules. Overall, eight CHOs provided either optional or mandatory e-learning to any professional group. In five CHOs (25%), optional e-learning was available to all staff groups; e-learning completion was mandatory for at least one staff group in three CHOs, and mandatory for all staff groups in only one.

## 3. Discussion

The findings of this survey showed a variable implementation of the national AMS toolkits in community healthcare organisations. Encouragingly, awareness of the TARGET and SSTF toolkits was high amongst the responding CHOs (90% and 75% of the responding CHOs, respectively) and 16 CHOS (80%) had either informally or formally reviewed either toolkit. This level was similar to that seen previously in secondary care acute trusts (87%) [11]. However, implementation of both toolkits (for instance formal review and implemented audits) was evident in under half of CHOs. Work in primary and secondary care found that having formally or informally reviewed AMS toolkits was associated with the development of action plans or the implementation of the toolkits [11]. This may also be the case in the current study. Two trusts without formal/informal review of the TARGET toolkit had also not implemented this toolkit, whereas implementation was evident (in the form of written action plans or implemented audits) for ~40–60% of CHOs who had reviewed the TARGET toolkit. Whether a formal/informal review of the SSTF toolkit led to toolkit implementation was impossible to ascertain.

There was little evidence to determine conclusively whether the implementation of either toolkit was influenced by CHO size or characteristics (the number of beds and bed type), although, in general, the level of implementation appeared higher amongst CHOs with over 200 beds.

Previous work has highlighted the potential importance of specialist antimicrobial pharmacists in delivering AMS activities [11]. However, only 25% of the responding CHOs had such a post. Whilst this figure was greater than that in primary care organisations (5%), it was much lower than that in secondary care (94%) [11]. In comparison to primary and secondary healthcare organisations, dedicated antimicrobial policies were only in place in 70% of the CHOs (99% and 93% in primary and secondary care, respectively). Equally, a lower proportion of CHOs (55%) had empirical antibiotic guidelines compared to primary care (73%) and secondary care trusts (83%). An AMS committee was active in 50% of the CHOs compared to 18% in primary care and 94% in secondary care [11]. Further research is evidently necessary to establish the relationship between the presence of AMS committees and/or dedicated AMS posts and the implementation of AMS toolkits, particularly in the CHO setting.

The results also showed that there was inadequate AMR/AMS education and training provided in CHOs. Half of the CHOs provided AMR/AMS teaching on induction to doctors, though only 40% provided this to both doctors and non-medical prescribers and 25% of the CHOs provided AMR/AMS induction training to all staff groups. The more frequent provision of AMS training to doctors and pharmacists compared to nurses and all other staff groups was also seen in secondary care [11] and reflects a pattern seen worldwide [13]. The code of practice states that all prescribers should receive AMR/AMS teaching on induction [9]. The improvement of ”professional education, training and public engagement” is also one of the seven key areas within the UK five-year AMR strategy [6], highlighting the importance of this area. Further effort is therefore required to promote delivery of AMS in CHOs; education and training can play an important role in helping to achieve this. With very limited specialist antimicrobial pharmacists in CHOs, regional medicines optimisation committees, NHS England/Improvement and PHE will need to work through senior pharmacists as well as medical and nursing directors to improve AMS activities in these settings. This could include promotion of the Health Education England introductory AMR e-learning module [14] and adaptation of the antimicrobial stewardship surveillance tools for use in community health organisations. In a similar way to primary and secondary care, it will be important to consider collation, analysis, and publication of antimicrobial consumption data in these settings to monitor the progress on improving prudent antibiotic use. 

Previous work has noted that the AMS focus on and audits relating to the ”start smart” aspects of SSTF were more common than those relating to “then focus” [11,15]. Further research would be helpful to look more in-depth at the implementation of AMS audits in CHO settings, i.e., data that was not captured in the current survey.

The major limitation of this study is that data were self-reported by pharmacists practising in CHOs; the authors could not verify that the reported AMS activities were actually taking place. Due to the low numbers, it was also difficult to assess the factors affecting successful implementation of the AMS toolkits.

A collaborative cross-sector approach to the delivery of AMS is in line with a move towards integrated care and is likely important in terms of optimising antibiotic usage across the healthcare system [11,16]. A health-system-wide perspective is also inherent to the national guidance on AMS [8]. Such collaboration between primary and secondary care in the UK has already been noted [9]. Given the cross-cutting position of CHOs within the healthcare sector, embedding of successful AMS strategies and toolkits in CHO settings will likely play a key role in promoting system-wide optimal antibiotic use. This highlights the importance of assessing AMS strategies within the CHO setting. To our knowledge, the current study is the first to do this. Further research will be necessary to assess, in greater depth, the factors affecting the successful implementation of AMS toolkits and to identify barriers to implementations in this setting.

## 4. Materials and Methods

A web-based cross-sectional survey based on previous studies for primary care and acute care organisations was developed using SelectSurvey. Six pharmacists working in Community Health Organisations (CHOs) piloted the survey in October 2015. The survey was subsequently revised and sent out to all representing CHOs in England during February 2016 using the community health services pharmacists’ network. The survey consisted of 50 items. In addition to general demographic questions and CHO information, the questions covered antimicrobial policies and guidance and prescribing support; the presence of AMS-dedicated staff posts and committees; awareness, implementation, and audit of AMS toolkits; and education in antimicrobial prescribing/stewardship. This survey (Supplementary Materials) was a voluntary service evaluation completed by healthcare professionals and thus ethical approval was not required. Survey responses were analysed using Microsoft Excel 2010 and Stata 13.

## 5. Conclusions

The findings of this survey showed that there was variable implementation of the national AMS toolkits (Start Smart then Focus and TARGET) in community healthcare organisations. The results also highlighted that the AMR/AMS education and training provided in CHOs may be inadequate. The findings from this study along with previous studies assessing AMS activities in primary and secondary care highlight the need for a cross-sector approach to the delivery of AMS.

## Figures and Tables

**Table 1:** Summary of antimicrobial stewardship activities and the implementation of national toolkits.

AMS Actions	Number (%) of CHOs (*N* = 20 CHOs Total)	
**AMS activities:**		
AMS committee active	10 (50%)	
AMS pharmacist on site	5 (25%)	
Grade of AMS pharmacy team:		
Pharmacy technician—band 4 or 5	0 (0%)	
Pharmacist—band 7	1 (5%)	
Pharmacist—band 8a	3 (15%)	
Pharmacist—band 8b	1 (5%)	
Remaining pharmacist posts	0 (0%)	
**Antibiotic policies in place:**		
Antimicrobial formulary	17 (85%)	
Empirical antibiotic guidelines	11 (55%)	
Restricted antibiotic list	2 (10%)	
Intravenous to oral switch	3 (15%)	
Surgical antimicrobial prophylaxis	2 (10%)	
Automatic stop	3 (15%)	
Separate antimicrobial drug chart/section	3 (15%)	
Outpatient parenteral therapy (OPAT)	10 (50%)	
**Awareness and implementation of AMS national toolkits:**		
	TARGET:	SSTF:
Awareness of toolkit	18 (90%)	15 (75%)
Toolkit formally reviewed	11 (55%)	10 (50%)
Toolkit-recommended audits implemented	10 (50%)	15 (75%)
Toolkit action plan in place	7 (35%)	8 (40%)

**Table 2:** Implementation of education and training initiatives in responding CHOs.

Healthcare Professional Groups	Percentage of CHO Organisations that Provide the Following Education and Training Initiatives to Healthcare Professionals			
	Doctors	Nurses	Pharmacist	Non-Medical Prescribers
Received AMS/AMR teaching on induction	50%	40%	50%	50%
Provided with antibiotic guidelines on induction	75%	55%	65%	75%
Have access to optional e-learning	25%	30%	25%	30%
Need to complete mandatory e-learning module	15%	10%	10%	10%

## Supplementary Materials

The survey tool used is available online at http://www.mdpi.com/2079-6382/7/4/97/s1.
