# Supplementary material for: Antimicrobial Stewardship Programmes in Community Healthcare Organisations in England: A Cross-Sectional Survey to Assess Implementation of Programmes and National Toolkits
Source: Antibiotics (Basel). 2018 Nov 7;7(4):97. doi: 10.3390/antibiotics7040097 (PMC6316239; doi:10.3390/antibiotics7040097)
Supplement: Supplementary file 1 [file antibiotics-07-00097-s001.pdf]

Public Health England

Antimicrobial Stewardship Survey for Community Health Services Trusts

You are invited to complete the following survey on antimicrobial stewardship (AMS) in Community Health Services (CHS) NHS Trusts.

The aim of the survey is to provide an understanding of current AMS practices in community hospitals and is the first step in developing a tool for community hospitals.

Implementation of AMS principles as demonstrated by national AMS toolkits, can help organisations to ensure compliance with the Health and Social Care Act 2008: Code of Practice on the prevention and control of infections and related guidance. Implementation also supports recommendations made in the NICE AMS guidance; Antimicrobial stewardship: systems and processes for effective antimicrobial medicine use (NG15), which was published in August 2015.

This survey is part of a PhD research project by Mary Akpan (University of Hertfordshire) in collaboration with Public Health England. The survey should take about 20 to 25 minutes to complete.

Thank you for your time.

(Please contact diane.ashiru-oredope@phe.gov.uk for any further information)

Public Health England

Antimicrobial Stewardship Survey for Community Health Services Trusts

About your hospital

1. Please select the name of your CHS Trust*

   -- Please Select --

2. Please provide the postcode of your CHS Trust (Main Office).*

3. Please provide your name*

   This information will not be published or shared with any third party.

4. Please provide your email address*

   This information will not be published or shared with any third party

5. What is your position in the CHS Trust?*

   - Head of Medicines Management
   - Clinical Pharmacist AfC 6/7
   - Clinical Pharmacist AfC 8a
   - Pharmacist AfC 8b/c
   - Other, please specify

6. How many community hospitals are managed by your CHS Trust?

7. Approximately, how many inpatient beds are there in your CHS Trust?

   -- None --

8. Which of the following services are offered by your CHS Trust?

   Please select all that apply

   - Addictions Management
   - Day Care Services (Social)
   - Day Surgery

- DayTreatments
- Dentistry
- Diagnostics
- Elective Surgery
- GP Services
- Learning Disabilities
- Maternity
- Mental health
- Minor Injury and Illness Centre
- Offender's Care
- Older Adults
- Outpatients
- Palliative Care
- Physiotherapy Services
- Podiatry
- Rehabilitation
- Sexual Health
- Walk in Centre
- Other, please specify

9. Please indicate the kinds of bed available in your CHS Trust.
Please select all that apply
- Inpatient step up beds
- Inpatient step down beds
- Mental health care beds
- Other, please specify

10. Which CCG commissions your CHS Trust (both inpatient and community)?*
-- Please Select --

Public Health England

Antimicrobial Stewardship Survey for Community Health Services Trusts

Antimicrobial Pharmacist

11. How many pharmacists work in your CHS Trust (whole time equivalents)?

12. Is there a substantive pharmacy post(s) focused on antimicrobial stewardship in your CHS Trust?*
- Yes
- No

13. How is the antimicrobial pharmacist post(s) graded?
Please select all that apply
- Pharmacy Technician - band 4
- Pharmacy Technician - band 5
- Pharmacist - band 6
- Pharmacist - band 7
- Pharmacist - band 8a
- Pharmacist - band 8b
- Pharmacist - band 8c
- Consultant Pharmacist
- Other, please specify

14. How much time in whole time equivalents (WTE) is dedicated to antimicrobial stewardship per week?
- ≤ 0.1
- 0.2 - 0.3
- 0.4 - 0.7
- 0.8 - 1.0
- > 1.0
- Other, please specify

15. You've indicated that there isn't a substantive pharmacy post focused on antimicrobial stewardship in your CHS Trust.
Does anyone else therefore have remit for antimicrobial stewardship?*
- Yes
- No
- Don't know

16. Please state who has remit for antimicrobial stewardship (job title of the postholder)?

17. What is the AfC band of the postholder?*

- Band 6
- Band 7
- Band 8a
- Band 8b
- Band 8c
- Band 9
- Other, please specify

18. How much time in whole time equivalents (WTE) is dedicated by the postholder to antimicrobial stewardship?

- ≤ 0.1
- 0.2 - 0.3
- 0.4 - 0.7
- 0.8 - 1.0
- > 1.0
- Other, please specify

Public Health England

Antimicrobial Stewardship Survey for Community Health Services Trusts

Your Antimicrobial Policy

19. Does your CHS Trust have a dedicated antimicrobial policy/management code, which details your overarching strategies for ensuring appropriate antimicrobial usage?*

- Yes
- No
- Don't know

20. If yes, when was it put in place?

21. When was the antimicrobial policy last reviewed?

22. How often is the antimicrobial policy reviewed?

- Twice yearly
- Once a year
- Every 2 years
- Every 3 years +
- Never
- Other, please specify

Public Health England

Antimicrobial Stewardship Survey for Community Health Services Trusts

Antimicrobial Stewardship

23. Does your CHS Trust have an antimicrobial stewardship committee/group?*

- Yes
- No

24. What professional groups are represented by your antimicrobial stewardship committee?

Please select all that apply

- CAMH Specialist
- Elderly Care Physician
- General Physician (GP)
- Junior Doctor
- Infectious Disease Specialist
- Mental Health Specialist
- Neurologist
- Paediatrician
- Specialist Physician
- Chief Pharmacist
- General Pharmacist
- Specialist Antimicrobial Pharmacist

- Infection Control Nurse
- Nurse
- CCG Representative
- Hospital Manager/Matron
- Microbiologist
- Podiatrist/Chiropodist
- Don't know
- Not Applicable
- Other, please specify

25. Do you have laymember representation in your antimicrobial stewardship committee?
- Yes
- No
- Don't know

26. Does the antimicrobial stewardship committee/group in your CHT Trust have the following?

	Yes	No	Don't know
Terms of Reference			
Minutes or Action Lists			
Minutes which go to Clinical Governance/Infection Control/drug & therapeutic committee			

27. You've indicated that your CHS Trust does not have an antimicrobial stewardship committee/group. Please indicate which committee(s) therefore has the written remit for antimicrobial stewardship in your CHS Trust?
Please select all that apply
- Infection Prevention and Control Commoittee
- Drugs and Therapeutics Committee
- Medicine Management Committee
- Don't know
- None
- Other, please specify

28. Which of the following guidelines/policies are in place in your CHS Trust?*
Please select all that apply
- Antimicrobial Formulary
- Empirical Antibiotic Guidelines
- Reserved Antimicrobial List
- Intravenous to oral switch
- Surgical Antimicrobial Prophylaxis Guidelines
- Automatic Stop Policy
- Separate Antimicrobial Drug Chart/Section
- Out-patient Parenteral Therapy (OPAT)
- Don't know
- Other, please specify

29. Who are the prescribers in your CHS Trust?*
Please select all that apply
- Consultants
- General Practitioners
- Junior Doctors
- Mental Health Doctors
- Nurses
- Podiatrists/Chiropodists
- Optometrists
- Physiotherapists
- Pharmacists
- Other, please specify

30. Which of the following applies to the prescribers in your CHS Trust?
Please select all that apply
- They are CHS Employees
- They are contracted to come, review and prescribe for your patients
- Don't know
- Other, please specify

Public Health England

Antimicrobial Stewardship Survey for Community Health Services Trusts

National Antimicrobial Stewardship Guidelines

31. Are you aware of the national AMS tookit TARGET (Treat Antibiotics Responsibly, Guidance, Education, Tools)?*
- Yes
- No

32. Please indicate what actions have been taken by your CHS Trust with regards to the national AMS toolkit TARGET?

*

	Yes	No	Don't know
Informally reviewed			
Formally reviewed			
Written action plan in place within antimicrobial policy/management code			
Action plan implemented within Antimicrobial policy/management code			
Implemented audits suggested by the audit plan within the national AMS toolkit			
Data collated as part of organisation-wide point prevalence surveys(PPS)			
Promote the use of the national AMS toolkit to prescribers for CPD/revalidation			
Promote the use of the national AMS toolkit within practice prescribing visits or teaching sessions			

33. Are you aware of the national AMS tookit SSTF (Start Smart Then Focus)?*
- Yes
- No

34. Please indicate what actions have been taken by your CHS Trust with regards to the national AMS toolkit SSTF?

*

	Yes	No	Don't know
Informally reviewed			
Formally reviewed			
Written action plan in place within antimicrobial policy/management code			
Action plan implemented within Antimicrobial policy/management code			
Implemented audits suggested by the audit plan within the national AMS toolkit			
Data collated as part of organisation-wide point prevalence surveys(PPS)			
Promote the use of the national AMS toolkit to prescribers for CPD/revalidation			
Promote the use of the national AMS toolkit within practice prescribing visits or teaching sessions			

35. Are you aware of the NICE Guidance: Antimicrobial Stewardship: Systems and processes for effective antimicrobial medicine use (NG15, August 2015)?*
- Yes
- No
- Don't know

36. Have you completed the baseline assessment tool which accompanies the NICE AMS guideline?*
- Yes
- No
- Don't know

37. If you have answered 'NO' to any of the relevant questions within the baseline assessment tool, have you developed an action plan?*
- Yes
- No
- Don't know

38. Has this action plan been implemented?*
- Yes
- No
- Don't know

39. In your CHS Trust, is it mandatory to document indication, duration, review and stop dates of antibiotics on drug charts?

	Yes	No	Don't know
Indication			
Patient's allergy status			
Treatment Duration			
Review Date			
Stop Date			

Public Health England

Antimicrobial Stewardship Survey for Community Health Services Trusts

Assessment of Effectiveness of Antimicrobial Stewardship

40. Which of the following outcomes are used to assess antimicrobial stewardship in your CHS Trust? *

Please select all that apply

- [ ] Total quantity of antimicrobial use in DDD (defined daily dose)
- [ ] Quantity of targeted antimicrobials in DDD
- [ ] Cost savings from all antibiotics
- [ ] Cost savings from targeted antibiotic class
- [ ] Percentage/number of multi-drug resistant organisms (MRSA, ESBL, Pseudomonas etc)
- [ ] Percentage/number of organisms resistant to certain antimicrobials
- [ ] Rate of C. difficile infection
- [ ] Clinical cure/improvement
- [ ] Rate of adverse antimicrobial reactions and interactions
- [ ] Other, please specify

41. Have you collected patient level audit/quality improvement data relating to antimicrobial stewardship within your CHS Trust in the last year?*

- ( ) Yes
- ( ) No
- ( ) Don't know

42. How frequently were audits/surveys/data collection on hospital antimicrobial stewardship carried out in the past year (Jan-Dec 2015)?

- ( ) Weekly
- ( ) Monthly
- ( ) Quaterly
- ( ) Twice a year
- ( ) Yearly
- ( ) Don't know
- ( ) Other, please specify

43. Please provide details of the audits which have been carried out in your CHS Trust?

Public Health England

Antimicrobial Stewardship Survey for Community Health Services Trusts

Education and Training

44. What antimicrobial prescribing and stewardship training takes place within the CHS Trust?

Please select to indicate whether the following antimicrobial and stewardship training takes place within your CHS. Blank = training does not take place

	Doctors	Nurses	Pharmacists	Non-Medical Prescribers
Teaching on induction	[ ]	[ ]	[ ]	[ ]
Antibiotic guidelines given	[ ]	[ ]	[ ]	[ ]
E-learning module optional	[ ]	[ ]	[ ]	[ ]
E-learning module mandatory	[ ]	[ ]	[ ]	[ ]
No training provided	[ ]	[ ]	[ ]	[ ]

45. Please provide information about any other antimicrobial prescribing and stewardship training which takes place in your CHS Trust.

46.

Select file to upload:

(click "Browse" button below to locate file)

File size restricted to: 3000 KB
File type restricted to: No file type restrictions.
Choose file No file chosen Upload

File Name: (limit 255 characters)

File Description: (limit 255 characters)

Files Uploaded:

Public Health England

Antimicrobial Stewardship Survey for Community Health Services Trusts

Clinical Decision Support

47. Does your CHS Trust have electronic prescribing for inpatients?*
    - Yes
    - No

48. Is there any clinical decision support about antimicrobial use built into any of the electronic prescribing software?*
    Clinical decision support = Electronic system to assist prescribers in the prescribing process and to therefore ensure correct and efficient prescribing of antimicrobials.
    - Yes
    - No

49. What kind of clinical decision support about antimicrobial use does the electronic prescribing software provide?
    Please select all that apply
    - Advice about the choice of antimicrobial
    - Advice about the dose of antimicrobial
    - Advice about the antimicrobial treatment duration
    - Trigger of patient's allergy status
    - Information about the cost of the antimicrobial
    - Information about whether or not the antimicrobial is on the formulary
    - Other, please specify

50. Does the CHS Trust currently work collaboratively with any of the following to deliver antimicrobial stewardship?
    Please select all that apply
    - Acute service provider
    - Other Community service provider
    - Private care provider
    - GP Practice
    - Mental Health provider
    - Ambulance service
    - Community Pharmacy
    - Urgent care provider
    - Out of hours provider
    - General Practice representation
    - Patient representation
    - Veterinary
    - Public Health - Local Authority
    - PHE Centres
    - Don't know
    - Other, please specify

51. Thank you very much for taking time to answer this survey. Please use this section if you would like to make any comments on this survey or the subject covered.
